# A R2R3-MYB Transcription Factor from *Epimedium sagittatum* Regulates the Flavonoid Biosynthetic Pathway

**DOI:** 10.1371/journal.pone.0070778

**Published:** 2013-08-01

**Authors:** Wenjun Huang, Wei Sun, Haiyan Lv, Ming Luo, Shaohua Zeng, Sitakanta Pattanaik, Ling Yuan, Ying Wang

**Affiliations:** 1 Key Laboratory of Plant Germplasm Enhancement and Specialty Agriculture, Wuhan Botanical Garden, Chinese Academy of Sciences, Wuhan, Hubei, China; 2 Key Laboratory of Plant Resources Conservation and Sustainable Utilization, South China Botanical Garden, Chinese Academy of Sciences, Guangzhou, Guangdong, China; 3 Department of Plant and Soil Sciences, University of Kentucky, Lexington, Kentucky, United States of America; University of Michigan, United States of America

## Abstract

Herba epimedii (*Epimedium*), a traditional Chinese medicine, has been widely used as a kidney tonic and antirheumatic medicine for thousands of years. The bioactive components in herba epimedii are mainly prenylated flavonol glycosides, end-products of the flavonoid pathway. *Epimedium* species are also used as garden plants due to the colorful flowers and leaves. Many R2R3-MYB transcription factors (TFs) have been identified to regulate the flavonoid and anthocyanin biosynthetic pathways. However, little is known about the R2R3-MYB TFs involved in regulation of the flavonoid pathway in *Epimedium*. Here, we reported the isolation and functional characterization of the first *R2R3-MYB* TF (*EsMYBA1*) from *Epimedium sagittatum* (Sieb. Et Zucc.) Maxim. Conserved domains and phylogenetic analysis showed that EsMYBA1 belonged to the subgroup 6 clade (anthocyanin-related MYB clade) of R2R3-MYB family, which includes *Arabidopsis* AtPAP1, apple MdMYB10 and legume MtLAP1. *EsMYBA1* was preferentially expressed in leaves, especially in red leaves that contain higher content of anthocyanin. Alternative splicing of *EsMYBA1* resulted in three transcripts and two of them encoded a MYB-related protein. Yeast two-hybrid and transient luciferase expression assay showed that EsMYBA1 can interact with several bHLH regulators of the flavonoid pathway and activate the promoters of dihydroflavonol 4-reductase (*DFR*) and anthocyanidin synthase (*ANS*). In both transgenic tobacco and *Arabidopsis*, overexpression of *EsMYBA1* induced strong anthocyanin accumulation in reproductive and/or vegetative tissues via up-regulation of the main flavonoid-related genes. Furthermore, transient expression of *EsMYBA1* in *E. sagittatum* leaves by *Agrobacterium* infiltration also induced anthocyanin accumulation in the wounded area. This first functional characterization of R2R3-MYB TFs in *Epimedium* species will promote further studies of the flavonoid biosynthesis and regulation in medicinal plants.

## Introduction

Flavonoids are a large group of diverse plant secondary metabolites that are derived from phenylalanine and malonyl-coenzyme A, including anthocyanins (red to purple pigments), flavonols (colorless to pale pigments) and proanthocyanins (PAs, also known as condensed tannins) that accumulate in a wide variety of plant tissues [1]. Flavonoids have a wide range of biological functions, including the attraction of pollinators and seed dispersers, and protection against UV light damage and pathogen attack [1], [2]. In recent years, research on flavonoids has been highly intensified due to their potential significant benefits on human health, including protection against cancer, cardiovascular diseases, inflammation and other age-related diseases [2], [3].

The flavonoid biosynthetic pathway is one of the most extensively studied pathways of plant secondary metabolites [4], [5]. The main structural genes encoding enzymes involved in this pathway have been isolated and characterized from many species, including *Arabidopsis*, maize, petunia, snapdragon, apple and grape [1], [6]–[8]. In plants, the structural genes of the flavonoid biosynthetic pathway are largely regulated at the level of transcription. It is well established that, in regulation of the flavonoid biosynthesis and cell fate, certain MYB TFs interact with bHLH TFs and WD40 proteins to form a MYB-bHLH-WD40 (MBW) complex [5], [9]. For example, the maize *MYB* gene (*ZmC1*) regulates the anthocyanin pathway by interacting with a *bHLH* partner (*ZmR* or *ZmB*) to activate the *DFR* (*ZmA1*) promoter [10].

MYB proteins, which comprise one of the largest TF families in the plant kingdom [11], are characterized by the highly conserved MYB DNA-binding domain (MYB domain). MYB family members are divided into four subfamilies, including 1R-, R2R3-, 3R-, and 4R-MYB proteins, depending on the number of MYB domains [12], [13]. Of the *MYB* genes identified in *Arabidopsis*, the 125 *R2R3-MYB* genes are most abundant [13]. A number of plant MYB TFs regulating the phenylpropanoid biosynthetic pathway have been identified from many species, including *Arabidopsis*, apple, grape, maize, petunia and snapdragon, most of which are R2R3-MYB TFs [14]. *MYB* regulators of the anthocyanin biosynthetic pathway have also been identified from many species, exemplified by *Arabidopsis MYB75* (*PAP1*) and *AtMYB90* (*PAP2*) [15], petunia *AN2* [16], grape *MYBA1* and *MYBA2* [17]–[19], sweet potato *MYB1* [20], apple *MYB10*/*MYB1*/*MYBA* [21]–[23], and legume *LAP1* [24].

*MYB* TFs have been proposed to generally regulate only one branch of the flavonoid pathway [14]. In *Arabidopsis,* for example, *AtTT2* and other *MYB* genes, including *Lotus japonicus TT2*, *Vitis vinifera MYBPA1* and *VvMYBPA2*, and *Diospyros kaki MYB4*, regulate PA biosynthesis [25]–[29], while *AtMYB12* and *VvMYBF1* regulate flavonol biosynthesis [30]–[32]. However, overexpression of *VvMYB5a* and *VvMYB5b* in tobacco has been reported to affect the entire phenylpropanoid pathway and metabolism of anthocyanins, PAs, flavonols and lignins [33], [34]. While most *R2R3-MYB* regulators of the flavonoid biosynthetic pathway have been demonstrated to be transcriptional activators, several *MYB* genes, including strawberry *FaMYB1* [35], snapdragon *AmMYB308* [36], and *Arabidopsis AtMYB4* as well as the single MYB-repeat *AtMYBL2* [37]–[39], have been identified as repressors.

Herba epimedii, a popular traditional Chinese medicinal plant, is derived from the dried aerial parts of *Epimedium* species (Berberidaceae family) widely distributed in China [40]. *E. sagittatum* (Sieb. et Zucc.) Maxim, together with four other *Epimedium* species, *E. brevicornu* Maxim, *E. pubescens* Maxim, *E. wushanense* T. S. Ying, and *E. koreanum* Nakai, is recorded in the Chinese Pharmacopoeia [41]. Herba epimedii contains various bioactive components, most of which are prenylated flavonoids, and has been used, in China, extensively as a kidney tonic and antirheumatic medicinal herb for thousands of years [42]. Currently, herba epimedii is also widely used to treat many diseases such as sexual dysfunction, osteoporosis, cardiovascular disease and tumors [42], [43]. In addition, *Epimedium* species exhibit a wide range of flower color, varying from white, yellow to red, crimson and violet, and leaf shape, and thus they are also popular as garden plants, particularly in Japan, Europe and America.

Due to significant beneficial effects on human health, there has been extensive, in-depth research on pharmacological functions of various phytochemicals [42]–[44]. The main components in Epimedium, which contribute to various bioactivities, have been demonstrated to be prenylated flavonol glycosides, end-products of a flavonol branch of the flavonoid biosynthetic pathway [42], [45]. Compared with the abundant information about the phytochemical aspect of herba epimedii, the molecular aspect has lagged behind, particularly on flavonoid biosynthesis and regulation responsible for the production and distribution of bioactive components and anthocyanin pigments. Recently, we have developed an *E. sagittatum* EST database, accelerating the discovery of genes involved in the flavonoid pathway [46]. Subsequently, a number of key structural genes of flavonoid biosynthesis, isolated from *E. sagittatum*, are being functionally characterized.

Little is known about the regulation of the flavonoid biosynthetic pathway by R2R3-MYB TFs at the transcriptional level in herba epimedii. Here, we report the functional characterization of a *R2R3-MYB* transcriptional regulator, *EsMYBA1*, isolated from *E. sagittatum*. *EsMYBA1* shares a high level of sequence homology and genomic structure with other plant *R2R3-MYB* genes involved in regulation of the anthocyanin biosynthesis. Alternative splicing of the *EsMYBA1* gene produces three transcripts, encoding a R2R3-MYB or a MYB-related protein. In addition, *EsMYBA1* is preferentially expressed in leaves of Epimedium. Both yeast two-hybrid and transient luciferase assay showed that EsMYBA1 interacts with several heterologous or homologous bHLH TFs known to be involved in regulation of the flavonoid pathway. Overexpression of *EsMYBA1* in tobacco and *Arabidopsis* up-regulates most of the flavonoid genes and greatly induces anthocyanin accumulation. Furthermore, *in vitro* transient expression of *EsMYBA1* also induces anthocyanin accumulation in the wounded area of leaves of *E. sagittatum*.

## Materials and Methods

### Plant Materials

Plants of *Epimedium sagittatum* were transplanted from Hunan province, China and grown in the experimental field of the Epimedium repository at Wuhan Botanical Garden in China. *Arabidopsis thaliana* ecotype Columbia, *Nicotiana tabacum* and *N. benthamiana* were grown in a glasshouse until required.

### DNA and RNA Extraction

Genomic DNA from young leaves of *E.sagittatum* was isolated with DNAquick plant system kit (Tiangen, China). Total RNA was isolated using RNAiso Plus (Takara, Japan) from several tissues of *E.sagittatum*, including leaf, petiole, flower bud and flower. For RNA extraction from fruit and roots, RNAiso-mate for plant tissue (Takara, Japan) was combined together with RNAiso Plus. The RNA solution was digested with RQ1 RNase-Free DNase (Promega, USA) to remove any contaminating genomic DNA before reverse transcription. Quality and quantity of nucleic acids was measured using a NanoDrop 2000c spectrophotometer (Thermo Scientific, USA).

### Isolation of *EsMYBA1* cDNA

The conserved R2 and R3 MYB domains of Epimedium *MYB* cDNA was obtained by PCR from first strand leaf cDNA with degenerate primers (listed in the Table S1 in File S1) which were designed based on highly conserved regions of previously isolated *R2R3-MYB* TFs known to regulate the anthocyanin biosynthesis in plants. The single PCR product obtained was cloned into the pMD19-T vector (Takara, Japan) and then sequenced. To obtain the corresponding full-length cDNA clone, Rapid Amplification of cDNA Ends (RACE) technology was adopted with SMART RACE cDNA Amplification kit (Takara, Japan). The full-length cDNA clone was isolated with primers (listed in Table S1 in File S1) and PrimeSTAR HS DNA Polymerase (Takara, Japan), and then designated as *EsMYBA1* (*Epimedium sagittatum MYB Anthocyanin-related* 1) gene, encoding a *R2R3-MYB* TF. Interestingly, two additional weak bands were observed when the expected main band was amplified. Through sequencing, these two cDNA clones were identified as alternative splicing transcripts of *EsMYBA1* gene, and they contained intron I and intron II and were designated as *EsMYBA1.1* and *EsMYBA1.2*, respectively. PrimeSTAR HS DNA Polymerase (Takara, Japan) and the same set of primers used for full-length cDNA amplification were used to amplify the genomic clone of *EsMYBA1* from genomic DNA of Epimedium leaves. The full-length cDNA and DNA sequences of *EsMYBA1* have been deposited in the GenBank database with the accession number KC335202 and KC335203, respectively.

### Quantitative RT-PCR

Quantitative RT-PCR (qRT-PCR) was used to determine the mRNA expression levels of the *EsMYBA1* gene in Epimedium tissues and the flavonoid-related genes in transgenic tobacco and *Arabidopsis*. Total RNA was extracted from various tissues of Epimedium as described above, while total RNA was isolated from transgenic tobacco flowers and leaves, and *Arabidopsis* seedlings using RNAiso plus (Takara, Japan). One microgram of total RNA was reverse transcribed with a PrimeScript RT reagent kit and gDNA eraser (Takara, Japan) was used to remove any contaminating genomic DNA. Quantitative PCR (qPCR) assay was performed using SYBR Premix Ex Taq II kit (Takara, Japan) and run on an ABI7500 Real-Time PCR machine (ABI, USA) following the manual’s recommendations. Gene specific primers for qPCR assay of Epimedium, tobacco and *Arabidopsis* were listed in Tables S1, S2, and S3 in File S1, respectively. After the end of the qPCR program, melting curve analysis was performed to ensure amplification of specific products. The comparative Ct method was used to determine the relative expression level.

### Subcellular Localization

The ORF (open reading frame) of EsMYBA1 (without stop codon) was amplified with primers listed in Table S1 in File S1 and cloned into the pBI221-GFP vector to create a CaMV 35S: MYBA1-GFP fusion construct which was bombarded into onion epidermal cells using Biolistic PDS-1000 (Bio-Rad, USA) for subcellular localization analysis. Samples were observed with confocal laser microscope and compared to the control expressing the pBI221-GFP empty vector.

### Yeast Two-hybrid Assay

In order to detect the interaction of EsMYBA1 with bHLH TF known to be involved in regulating the anthocyanin biosynthetic pathway, yeast two-hybrid (Y2H) assay was performed as previously described [47]. The plasmids pAD-GAL4-2.1 and pBD-GAL4-Cam (Stratagene, USA), containing the GAL4 activation and GAL4 DNA-binding domains, respectively, were used. The full-length coding region of EsMYBA1 was cloned into both pAD-GAL4 and pBD-GAL4 vectors. The BD-bHLH constructs contain the MYB-interaction domain (ID) of seven plant bHLH TFs, including maize Lc^aa1−212^, snapdragon Delila^aa1−201^, perilla Myc-Rp^aa1−199^, *Arabidopsis* GL3^aa1−209^ and TT8^aa1−204^, and tobacco AN1a^aa1−195^ and AN1b^aa1−195^, fused with the GAL4 DNA-binding domain [47], [48]. Both pAD-EsMYBA1 and pBD-bHLHs plasmids were co-transformed into yeast strain AH109 using the PEG/LiAC method. Co-transformation of pAD-EsMYBA1 and pBD-GAL4 empty vector was used as negative control. Transformed colonies were selected on synthetic drop-out medium lacking leucine and tryptophan (SD-Leu-Trp). Colonies from double selection plates were then screened for growth on quadruple selection SD medium lacking adenine, histidine, leucine and tryptophan (SD-Ade-His-Leu-Trp).

### BiFC Assay in *Arabidopsis* Mesophyll Protoplasts

For BiFC (bimolecular fluorescent complementation) assay, we used expression vectors pNYFP and pCYFP, containing the N- and C-terminal halves of yellow fluorescent protein (YFP), respectively, gifted from Professor Ling Yuan of the University of Kentucky [47]. For the generation of BiFC vectors, the full-length coding sequence of *EsMYBA1* was cloned into pNYFP as a *Xho*I-*Bam*HI fragment to form the EsMYBA1-NYFP construct. The NtAN1a-CYFP construct, containing the full-length coding sequence of *NtAN1a* (GenBank accession number: HQ589208) fused with a C-terminal fragment of YFP, was provided by the laboratory of Ling Yuan [48]. Expressions of *EsMYBA1* or *NtAN1a* alone were used as negative controls. The resulting constructs were used for transient assays by polyethylene glycol (PEG) transfection of *Arabidopsis* protoplasts isolated from 4-week-old wild-type Columbia plants according to previously reported procedures [49]. mCherry-VirD2NLS was induced in each transfection to serve as a control for successful transfection as well as for nuclear localization [50]. Transfected cells were imaged using a confocal microscope. The primers used for BiFC assay are also listed in Table S1 in File S1.

### Transient Luciferase Assay of *EsMYBA1* against Promoters of Anthocyanin Biosynthetic Genes

Transcription activity of EsMYBA1 TF against promoters of anthocyanin biosynthetic genes was performed using dual luciferase assay of transiently transformed *N. benthamiana* leaves [51]. A 1277 bp *AtDFR* promoter (Accession number: AT5G42800) from *A. thaliana* and a 566 bp *NtDFR* promoter (Accession number: FJ472649) from *N. tabacum* were amplified, respectively. Both 5′-flanking regions of *EsDFR* and *EsANS* from *E. sagittatum* were isolated by Tail-PCR (thermal asymmetric interlaced PCR) and sequenced. A 1429 bp *EsDFR* promoter (Accession number: KC335205) and a 1566 bp *EsANS* promoter (Accession number: KC335207) were amplified from genomic DNA of *E. sagittatum*, respectively. All primers used for promoter sequence isolation are listed in Table S1 in File S1. Promoters were subcloned into the transient expression reporter vector pGreenII 0800-LUC which contains the CaMV 35S promoter-REN cassette and the promoterless-LUC cassette [51]. Effector constructs were generated by subcloning coding regions of *EsMYBA1*, *EsTT8* (Accession number: KC686401) and *AtTT8* (Accession number: NM_117050) TFs into the transient expression vector pGreenII 62-SK which contains the CaMV 35S promoter-MCS-CaMV terminator cassette, using primers listed in Table S1 in File S1 [51]. In addition, *EsTT8* gene, encoding a *bHLH* TF, was isolated from *E. sagittatum* with primers in Table S1 in File S1. *Agrobacterium-*infiltrated transient transformation of *N. benthamiana* was carried out as previously described [51]. In brief, *N. benthamiana* plants were grown under glasshouse conditions until about 5 cm in height. Approximately, 300 µl of *Agrobacterium* containing the reporter or/and effector plasmids was infiltrated into a young leaf at two points and transient expression was assayed after three days of inoculation. Firefly luciferase and renilla luciferase were assayed using the dual luciferase assay reagents (Promega, USA). Data was collected as the ratio of LUC/REN. Background controls were run with only the reporter construct. At least four plants at the same developmental stage were used for each treatment, and the experiment was repeated three to four times.

### Overexpression Vector Constructs and Plant Transformation

For plant transformation, the full-length cDNA of *EsMYBA1* was transferred from pMD19-T vector (Takara, Japan) digested with *Sal*I and *Kpn*I, to the modified binary pMV vector, derived from the pBI121 vector, digested with *Xho*I and *Kpn*I, resulting in the pMV-EsMYBA1 construct. This construct, containing the *EsMYBA1* cDNA under the CaMV 35S promoter and nos terminator, was introduced into *Agrobacterium tumefaciens* strain EHA105 or GV3101 by electroporation and then used for *A. thaliana* (Columbia ecotype) and tobacco transformation. *Agrobacterium*-mediated transformation of *Arabidopsis* was performed using floral dip method [52], and tobacco transformation was carried out using leaf disc method [53]. Transformed plants were selected using kanamycin (100 µg/mL) as a plant selective marker and the presence of transgene was detected by PCR. Four independent T_0_ transgenic tobacco plants and two T_2_ transgenic *Arabidopsis* lines showing obvious phenotypic changes were used for further analysis. For transient expression of *EsMYBA1* in Epimedium, *Agrobacterium*-mediated transformation of Epimedium was performed as described for tobacco transformation and pigmentation was observed after 3 days of co-culture. Transgenic plants expressing the pMV empty vector were used as negative controls in the plant transformation of these three species.

### Determination of Total Anthocyanin Content

Total anthocyanin was extracted from various fresh tissues, using 1% HCl in methanol (v/v) in the dark at 4°C overnight with occasional shaking. The extracts were centrifuged at 10,000 g for 5 min and the supernatant was used for determination of absorbance at 530 nm and 657 nm. Total anthocyanin content was quantified using the equation (A530-0.25×A657)/fresh weight which compensates for the contribution of chlorophyll and its degradation products with absorbance at 530 nm [54]. Three replicates were analyzed for each sample.

## Results

### Isolation and Sequence Analysis of *EsMYBA1* Gene

Degenerate PCR primers, based on conserved residues of R2 and R3 MYB domains, amplified a 233-bp band from mRNA isolated from *E. sagittatum* leaves. The PCR products were cloned and transformed into *E. coli*, and a dozen independent clones were sequenced. All sequences shared >94% identity and yielded a single ORF. RACE experiments were carried out to isolate the full-length cDNA (FLC) clone, here designated as *EsMYBA1*. Based on a total of 24 of FLC sequencing results, three different transcripts were identified, of which two abnormal transcripts, designated as *EsMYBA1.1* and *EsMYBA1.2*, contained intron I and II, respectively. The *EsMYBA1* FLC used for functional analysis contains an ORF of 714 bp that encodes a *R2R3-MYB* TF comprised of 237 amino acids (aa). One additional *EsMYBA1* FLC was identified to have an ORF of 711 bp, encoding 236 aa, resulting from a three nucleotide deletion in the 3′-terminal region which corresponded to a single amino acid (E166) deletion. This copy of *EsMYBA1* has not been included in the present functional characterization.

**Figure 1:**
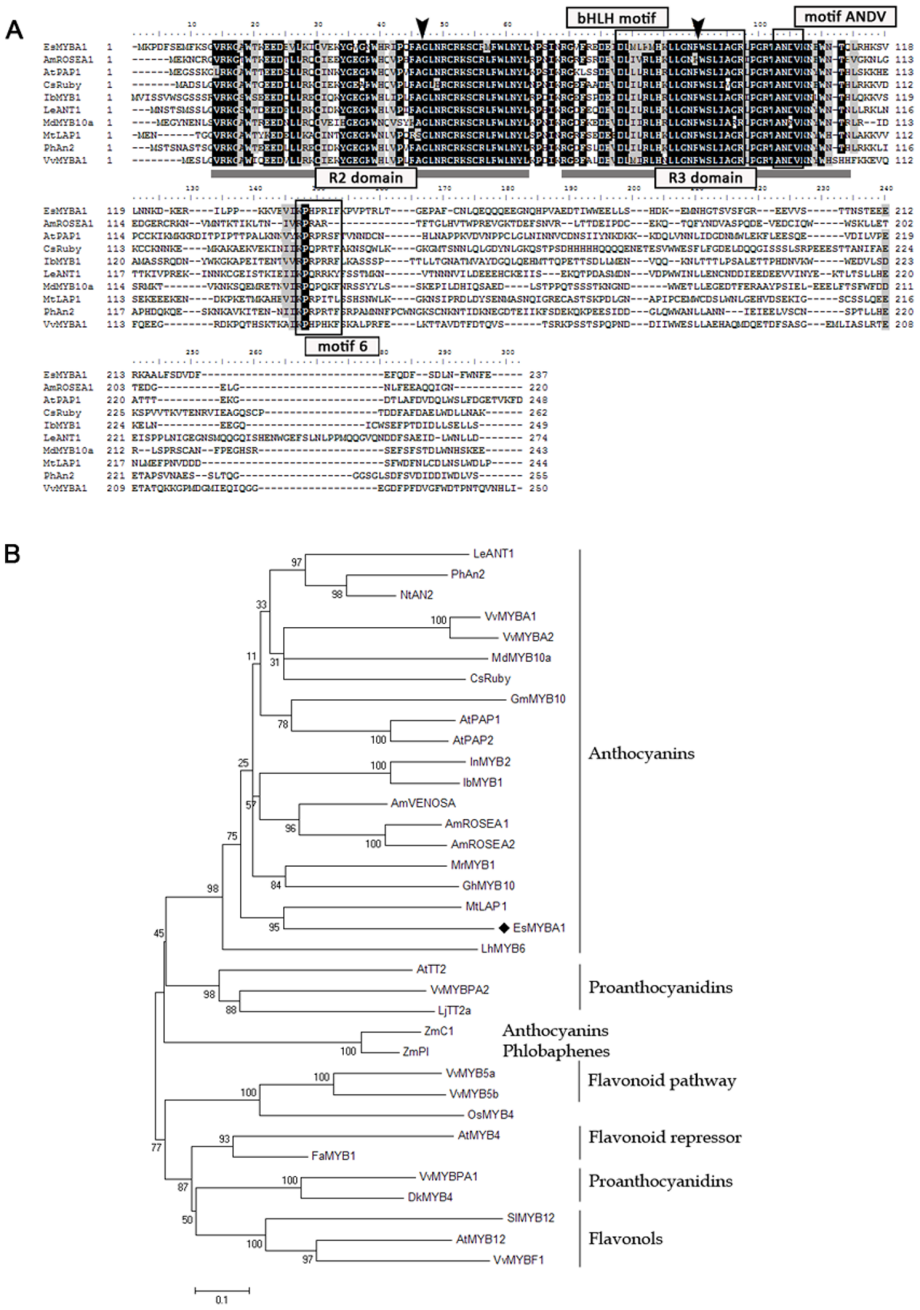
Multiple alignment and phylogenetic analysis of EsMYBA1 and related plant R2R3-MYB proteins known to regulate the flavonoid pathway. (A) Alignment of deduced amino acid sequences of EsMYBA1 and other plant R2R3-MYB proteins. Identical amino acid residues are shaded in black, similar in gray. The R2 and R3 domains shown refer to two repeats of the MYB DNA-binding domain of selected MYB proteins. Three conserved motifs, the bHLH interaction motif, the ANDV motif identified in Rosaceae and the motif 6 from *Arabidopsis* R2R3-MYB family classification are boxed. The two arrowheads indicate the insert position of intron I and II, respectively. (B) Phylogenetic tree of EsMYBA1 and selected R2R3-MYB proteins from other plant species using the neighbor-joining method by the MEGA 5 software. The scale bar represents the number of substitution per site and the numbers next to the nodes are bootstrap values from 1,000 replicates. The EsMYBA1 are indicated as a diamond. The putative regulatory functions of the different R2R3-MYB proteins in the control of phenylpropanoid biosynthesis pathway are indicated. All R2R3-MYB protein sequences were retrieved from GenBank database and accession numbers are as follows (in parentheses): *Antirrhinum majus AmROSEA1* (ABB83826); *AmROSEA2* (ABB83827); *AmVENOSA* (ABB83828); *Arabidopsis thaliana AtPAP1* (AAG42001); *AtPAP2* (AAG42002); *AtTT2* (NP_198405); *AtMYB12* (ABB03913); *AtMYB4* (NP_195574); *Citrus sinensis CsRuby* (AFB73913); *Diospyros kaki DkMYB4* (BAI49721); *Fragaria x ananassa FaMYB1* (AAK84064); *Garcinia mangostana GmMYB10* (ACM62751); *Gerbera hybrid GhMYB10* (CAD87010); *Ipomoea batatas IbMYB1* (BAF45114), *Ipomoea nil InMYB2* (BAE94709); *Lycopersicon esculentum* (*Solanum lycopersicum*) *LeANT1* (AAQ55181); *SlMYB12* (ACB46530); *Lilium hybrid LhMYB6* (BAJ05399); *Lotus japonicus TT2a* (BAG12893); *Malus x domestica MdMYB10a* (ABB84753); *Medicago truncatula MtLAP1* (ACN795410; *Morella rubra MrMYB1* (ADG21957); *Nicotiana tabacum NtAN2* (ACO52470); *Oryza sativa OsMYB4* (BAA23340); *Petunia x hybrida PhAn2* (AAF66727); *Vitis vinifera VvMYBA1* (BAD18977); *VvMYBA2* (BAD18978); *VvMYBPA1* (CAJ90831); *VvMYBPA2* (ACK56131); *VvMYBF1* (ACV81697); *VvMYB5a* (AAS68190); *VvMYB5b* (AAX51291); *Zea mays ZmC1* (AAA33482); *ZmPl* (AAA19819).

*EsMYBA1* encodes a *R2R3-MYB* TF that contains the highly conserved R2 and R3 MYB domains in the N-terminal region. Within the conserved R2R3 domains, EsMYBA1 shows high identity with other MYB regulators of anthocyanin biosynthesis, sharing 79% identity with *Garcinia mangostana* MYB10 and 74% identity with *Arabidopsis thaliana* PAP1. However, when considering the overall protein sequences, less homology was present, including 50% identity with *Citrus sinensis* Ruby and 48% identity with *Myrica rubra* MYB1. In addition to the conserved R2 and R3 MYB domains, EsMYBA1 contained another three conserved motifs in the C-terminal region, the conserved [DE]Lx_2_[RK]x_3_Lx_6_Lx_3_R motif critical for interaction with bHLH proteins [55], the conserved ANDV motif identified from MYB regulators of the anthocyanin pathway in Rosaceae [56], and the motif 6 KPRPR[ST]F which is highly conserved in the R2R3-MYB subfamily six of *Arabidopsis* as described previously [13] (Figure 1A). Phylogenetic analysis of EsMYBA1 was performed with other known MYB regulators controlling different secondary metabolite biosynthesis. The tree showed that R2R3-MYB TFs with similar function clustered together, and EsMYBA1 was grouped into the large anthocyanin-related MYB clade and located in the basal position of clade (Figure 1B).

### Genomic Structure and Alternative Splicing of *EsMYBA1*

**Figure 2:**
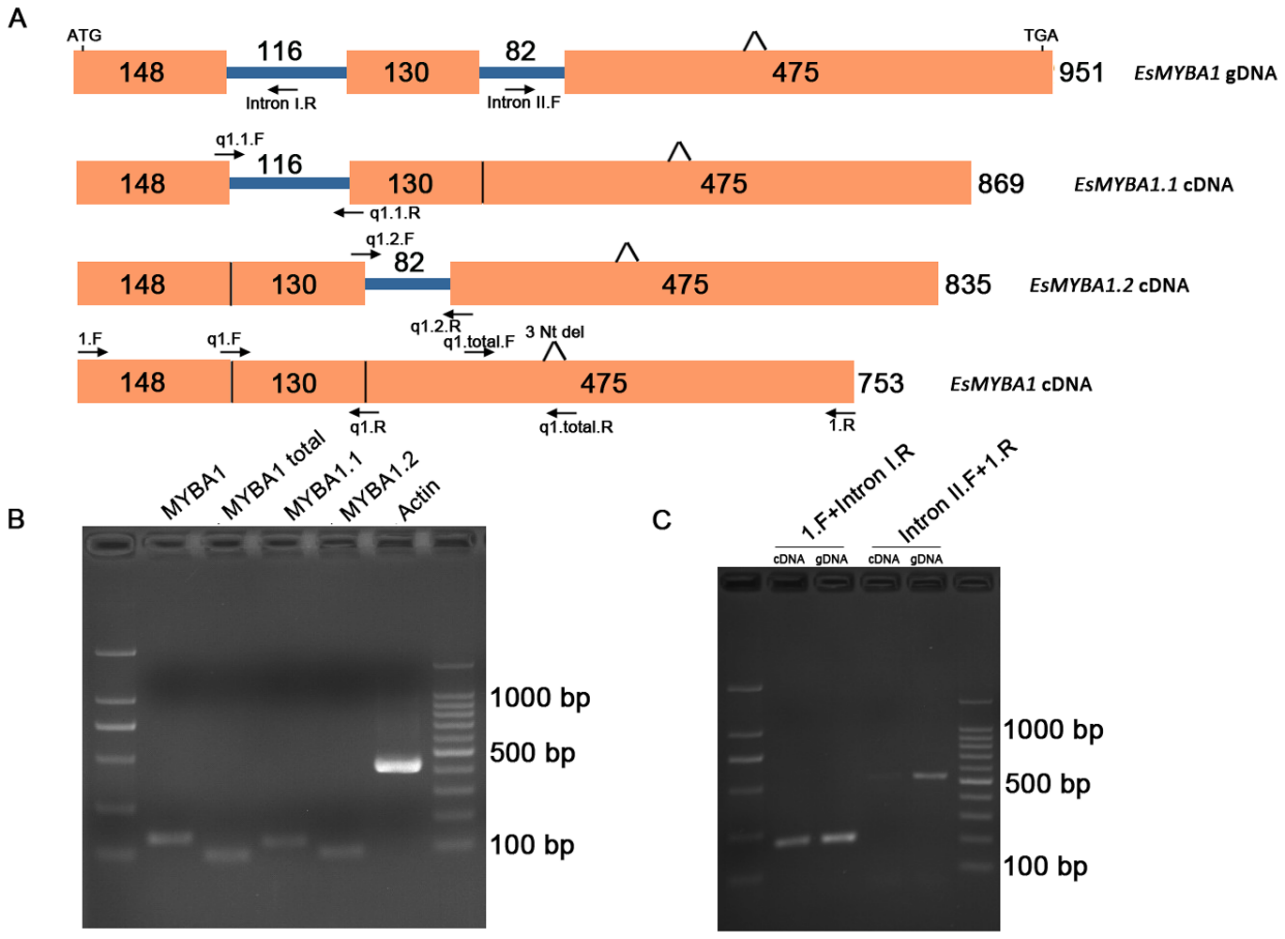
Genomic structure and alternative splicing analysis of *EsMYBA1* gene. (A) Schematic diagram of genomic structure and three different transcripts resulting from alternative splicing of *EsMYBA1* gene. The exons are shown as blocks and the introns as lines. The primers used in this study are shown as arrows and listed in the Table S1 in File S1. The three nucleotide deletion (Nt del) in both cDNA and gDNA sequences are indicated. Numbers refer to the fragment length from primer 1.F to 1.R used for the full-length *EsMYBA1* cDNA amplification. (B) Representative gel image of the amplicons corresponding to the different transcripts of *EsMYBA1* gene using the different transcript-specific primers and cDNA template from Epimedium leaves. Four amplicons, referring to the *EsMYBA1*, *EsMYBA1.1 EsMYBA1.2* and the specific fragment shared by these three transcripts (named as “*EsMYBA1* total”) are indicated, while the *Actin* gene of Epimedium is also indicated as a positive control. (C) Representative gel image of two amplicons amplification for confirming the alternative splicing of *EsMYBA1* gene. Two pairs of primers compassing the part intron I and intron II fragments, respectively, are used to amplify the two different fragments from both cDNA and gDNA templates. Each fragment from both cDNA and gDNA shows the same band size.

The genomic DNA (gDNA) sequence revealed several nucleotide mismatches with the cDNA sequence of *EsMYBA1.* Furthermore, the three nucleotide deletion was also found in the 3′-terminal region of the gDNA sequence (Figure 2A). Alignment analysis of cDNA and gDNA sequences revealed that the genomic structure of *EsMYBA1* consisted of three exons and two introns (Figure 2A). Alternative splicing was observed in the *EsMYBA1* gene. Introns I and II remained in two additional transcripts, designated as *EsMYBA1*.*1* and *EsMYBA1*.*2*, respectively (Figure 2A). Both *EsMYBA1*.*1* and *EsMYBA1*.*2* encode a truncated MYB protein with a partial or a single MYB repeat. Because of the presence of the unspliced introns, they were designated as MYB-related proteins [12]. Four pairs of gene specific primers (GSP), located at different transcript-specific positions, were designed for RT-PCR and qRT-PCR assay (Figure 2A). Using these GSPs, the amplicons corresponding to the three transcripts and their shared fragment (named as “*EsMYBA1* total”) were successfully amplified from leaf cDNA template (Figure 2B). In addition, two amplicons containing part of intron I and intron II fragments, respectively, were successfully amplified from both cDNA and gDNA (Figure 2C). These PCR results, together with sequencing analysis, demonstrated the alternative splicing for the *EsMYBA1* gene through intron retention.

### Expression Pattern of *EsMYBA1* in Various Tissues

**Figure 3:**
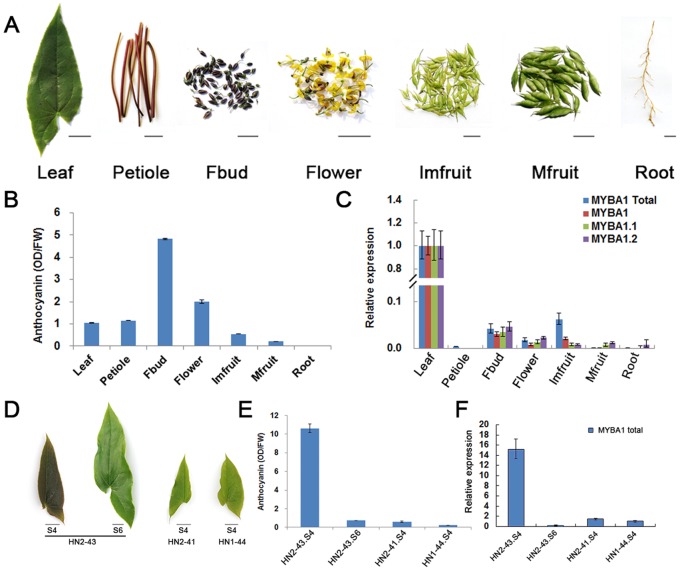
Quantitative RT-PCR analysis of *EsMYBA1* and total anthocyanin content measurement in various tissues of Epimedium. Photos of seven tissues from *E. sagittatum*, including leaf, petiole, flower bud (Fbud), flower, immature fruit (Imfruit), mature fruit (Mfruit) and root tissues (A), and four leaf samples from three plantlets of two populations of *E.sagittatum* at the two developmental stages (S4, fully opened young leaf with one-half size of mature leaf and S6, slightly leathered mature leaf) (D), bar = 1 cm. Total anthocyanin content from seven different tissues (B) and four leaf samples (E) above was measured. Each column represents the mean value with error bar indicating SD from three technical replicates for each sample. Quantitative RT-PCR analysis of different transcripts from *EsMYBA1* gene in seven tissues (C) and of “*EsMYBA1* total” in four leaf samples (F) was carried out. Four transcripts resulting from the alternative splicing of *EsMYBA1* gene, including *EsMYBA1*, *EsMYBA1.1*, *EsMYBA1.2* and “*EsMYBA1* total” (fragment shared by three transcripts, for detail explanation see Figure 2) were selected, and the *Actin* gene was used as an internal control. All primers used for qPCR analysis were listed in the Table S1 in File S1. The comparative Ct method was used to determine the relative level of gene expression. The column shows the average value with SD bar from three technical replicates.

To investigate whether the expression of *EsMYBA1* correlates with anthocyanin accumulation patterns in various tissues of Epimedium, qPCR assay was first used to determine mRNA levels isolated from seven tissues of the green-leafed *E. sagittatum* (Figure 3A). Anthocyanin accumulated most abundantly in red flower buds, abundantly in flowers, moderately in petioles and leaves, weakly in fruits, but not in roots (Figure 3B). However, there appeared to be no correlation between the *EsMYBA1* expression and anthocyanin accumulation in the green-leafed plants. Results from qPCR using transcript-specific primers indicated that the three transcripts had a similar expression pattern, expressing most highly in leaf, moderately in flower bud, weakly in flower and immature fruit, almost none in mature fruit, root and petiole (Figure 3C). We next compared the expression levels of “*EsMYBA1* total” and total anthocyanin content in green and red leaves of Epimedium. Four samples were collected from three plantlets of two populations of *E. sagittatum* at the two developmental stages (Figure 3D). In the young red leaf (HN2-43.S4), which contained the highest level of anthocyanin (Figure 3E), the expression level of “*EsMYBA1* total” was significantly higher than that in the other three green leaves at both S4 and S6 stages (Figure 3F).

### *EsMYBA1* is Predominantly Localized in Nucleus

**Figure 4:**
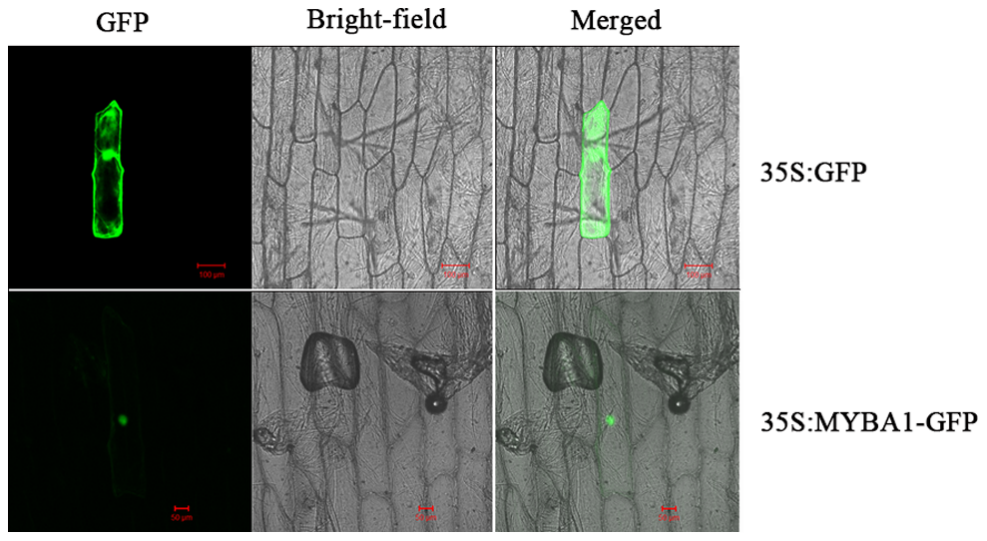
Subcellular localization of EsMYBA1 in onion epidermal cells. GFP and EsMYBA1-GFP fusion proteins were bombarded by gene gun and transiently expressed under control of the CaMV 35S promoter in onion epidermal cells and observed with a laser scanning confocal microscope. The length of the bar is indicated in the photographs.

To validate the subcellular localization of *EsMYBA1*, the coding region of *EsMYBA1* was fused in-frame to *GFP*, and the expression vector was delivered by gene gun for transient expression in onion epidermal cells. Compared with the distribution of GFP alone, fluorescence of EsMYBA1-GFP was predominantly localized in the nucleus (Figure 4).

### EsMYBA1 Interacts with bHLH Regulators of the Anthocyanin Biosynthetic Pathway

**Figure 5:**
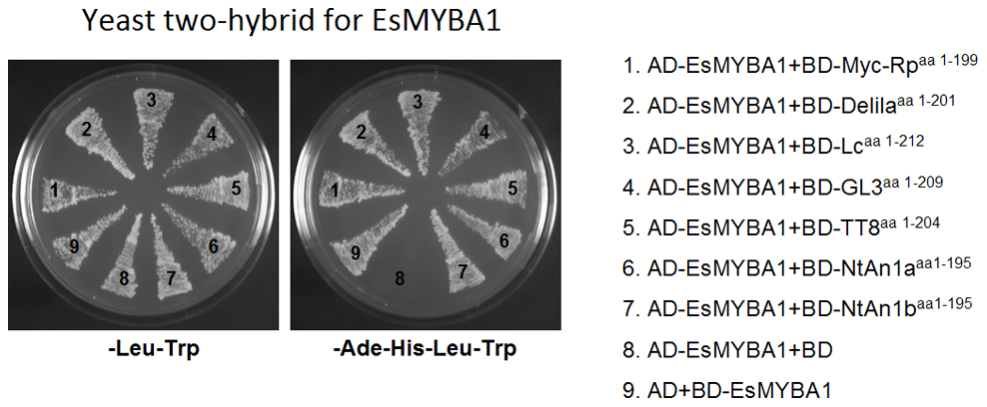
Physical interaction between EsMYBA1 and the MYB-interacting region (MIR) isolated from selected seven bHLH TFs detected in yeast two-hybrid assay. AH109 yeast strains were transformed with plasmids pBD-GAL4-EsMYBA1+ pAD-GAL4, pAD-GAL4-EsMYBA1+ pBD-GAL4, pAD-GAL4-EsMYBA1+ pBD-GAL4-MIR from selected seven bHLH TFs, including perilla *Myc*, snapdragon *Delila*, maize *Lc*, *Arabidopsis GL3* and *TT8*, tobacco *An1a* and *An1b*. The yeast transformants were grown in SD/-Leu/-Trp double (left) and SD/-Ade/-His/-Leu/-Trp quadruple (right) selection mediums.

To detect the interactive ability of EsMYBA1 with bHLH regulators of the anthocyanin pathway, Y2H was implemented for measuring the interaction between EsMYBA1 and the MYB-interacting domains isolated from seven flavonoid-related bHLH regulators, including perilla Myc, snapdragon Delila, maize Lc, *Arabidopsis* GL3 and TT8, and tobacco An1a and An1b. The autoactivation of EsMYBA1 was first investigated. Transformed yeast cells, harboring pBD-GAL4-EsMYBA1 (BD-EsMYBA1) plus pAD-GAL4 (AD), grew well on both double (SD/−Leu/−Trp) and quadruple (SD/−Ade/−His/−Leu/−Trp) selection medium, while the negative control, containing pAD-GAL4-EsMYBA1 (AD-EsMYBA1) and pBD-GAL4 (BD), did not grow on quadruple selection medium (Figure 5), indicating EsMYBA1 is capable of autoactivation. Subsequently, the AD-EsMYBA1 construct was co-transformed into yeast cells with the different BD-bHLH constructs. Yeast cells containing any one of seven combinations of EsMYBA1 plus bHLHs, grew well on both double and quadruple selection medium (Figure 5). The Y2H result demonstrated that EsMYBA1 not only could interact with these bHLH regulators of the flavonoid pathway, and also had the ability of self-activation.

**Figure 6:**
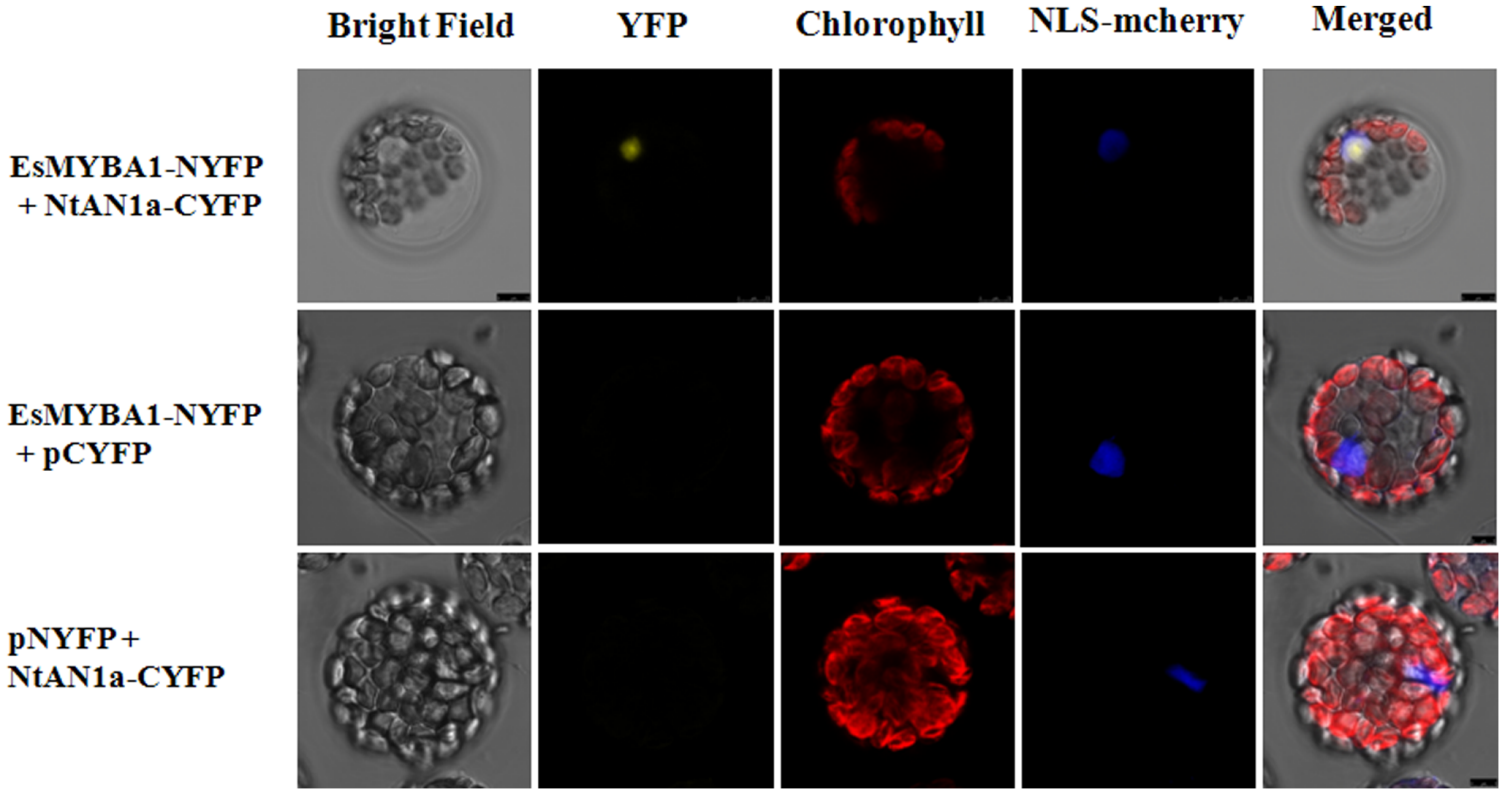
BiFC visualization of EsMYBA1 and NtAN1a interaction in transiently co-expressed *Arabidopsis* mesophyll protoplasts. EsMYBA1 protein was fused with the N-terminal half of YFP (EsMYBA1-NYFP) and NtAN1a protein was fused with the C-terminal half of YFP (NtAN1a-CYFP). The mCherry-VirD2NLS was induced in each transfection to serve as control for successful transfection as well as for nuclear localization. Two combinations of EsMYBA1-NYFP+pCYFP and NtAN1a-CYFP+pNYFP were used as negative controls.

We used a transient *Arabidopsis* protoplast BiFC assay to investigate whether the EsMYBA1-NtAN1a interaction observed in yeast cells also occurs in plant cells. A plasmid containing the N-terminal half of YFP fused to the EsMYBA1 cDNA (EsMYBA1-NYFP) and a plasmid harboring the C-terminal half of YFP fused to the NtAN1a cDNA (NtAN1a-CYFP) were transiently co-expressed in *Arabidopsis* leaf mesophyll protoplasts by PEG transfection. Protoplasts co-transfected with EsMYBA1-NYFP and NtAN1a-CYFP constructs produced a strong fluorescence signal that was localized in the nucleus (Figure 6). However, no fluorescence signal was observed when the two negative control combinations of EsMYBA1-NYFP+pCYFP and NtAN1a-CYFP+pNYFP were co-expressed in protoplasts (Figure 6). The BiFC results not only demonstrated the *in vivo* interaction between EsMYBA1 and NtAN1a, but also showed the specific localization of the interacting proteins in the nucleus.

### *EsMYBA1* Activates Promoters of Anthocyanin Structural Genes *DFR* and *ANS*

**Figure 7:**
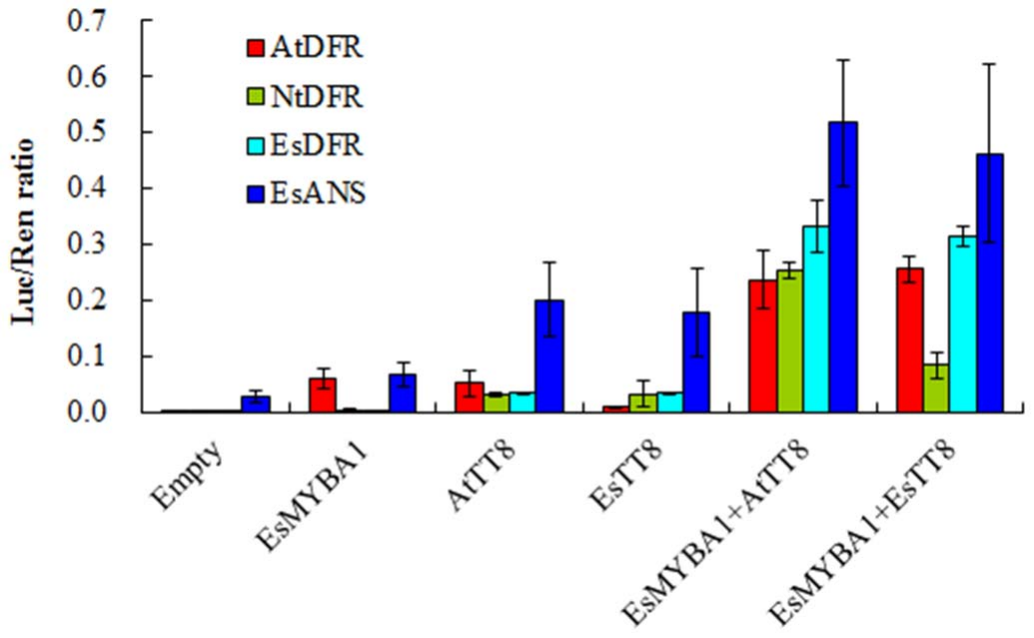
Interaction of EsMYBA1 and EsTT8, *Arabidopsis* TT8 TFs in a dual luciferase transient tobacco transformation assays affects the activity of the *DFR* promoter from *Arabidopsis*, tobacco and Epimedium, and the *ANS* promoter from Epimedium. Leaves of *Nicotiana benthamiana* were infiltrated with the reporter construct containing the *DFR* or *ANS* promoter-LUC fusions on their own (used as empty control) or co-infiltrated with the effector construct containing the *EsMYBA1*, *EsTT8* or *AtTT8* under control of CaMV 35S promoter alone or their combinations, and then luminescence of LUC and REN was measured 3 days later and expressed as a ratio of LUC to REN. Error bars are the SE for six replicate reactions.

Transient luciferase assays in *N.benthamiana* were used to determine EsMYBA1 activity, with or without bHLH TFs, against the *DFR* promoter from *Arabidopsis*, tobacco, and Epimedium, and the *ANS* promoter from Epimedium. Full-length cDNAs of EsMYBA1 and two bHLH TFs (*Arabidopsis* TT8 and Epimedium TT8) were cloned into the transient expression effector vector pGreenII 62-SK, and the *DFR* and *ANS* promoters were cloned into the reporter vector pGreenII 0800-LUC. The reporters and effectors were transformed into *Agrobacterium* and then co-infiltrated into *N. benthamiana* leaves. After 3 days, transactivation was quantified as a change in LUC/REN ratio. Generally, luciferase activity was noticeably enhanced when transformation was performed with a MYB or bHLH TF effector against all three *DFR* and one *ANS* promoters, compared with the no-effector control. These detectable activities are likely the results of the effectors interacting with an endogenous partner from *N. benthamiana*. However, these activities were significantly lower than those of co-transformation with both effector combinations (EsMYBA1+AtTT8 and EsMYBA1+EsTT8) (Figure 7). The individual TFs (EsMYBA1, EsTT8 and AtTT8) as effectors induced the *DFR* and *ANS* promoters to different extents, ranging from approximately 2 to 4 folds above the control. By comparison, the activation of *DFR* and *ANS* promoters was considerably enhanced, showing additional 2–4 fold increase compared to the single effectors, when both MYB and bHLH TF effectors were co-transformed (Figure 7). These results indicate EsMYBA1 can interact with EsTT8 or AtTT8 bHLH TFs in plant cells.

### Elevated Anthocyanin in Transgenic Tobacco and *Arabidopsis* Overexpressing *EsMYBA1*

**Figure 8:**
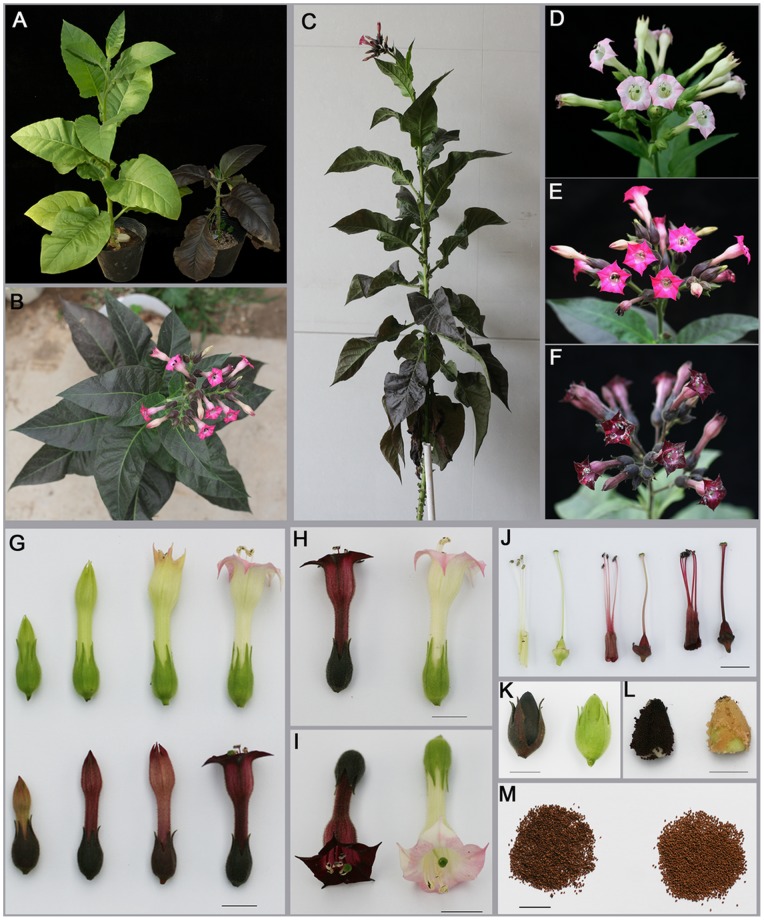
Phenotype observation of transgenic tobacco plants overexpressing *EsMYBA1* and empty vector (control). (A) Immature control (left) and *EsMYBA1*-expressing (right) plants. (B–C) Mature *EsMYBA1*-expressing plants at the blossoming stage from the different views. (D–F) Close views of control (D) and two *EsMYBA1*-expressing plants showing the strong (E) and extreme (F) color changes. (G) Flowers from control (top) and *EsMYBA1*-expressing (bottom) plants at the different developmental stage. (H–I) Intact flowers from control (right) and *EsMYBA1*-expressing (left) plants from the different views. (J) Dissected flowers showing stamen and pistil clearly from control (left) and two *EsMYBA1*-expressing (middle and right) plants. (K–M) Immature capsules (K) and immature seeds (L) and mature seeds (M) from control (right) and *EsMYBA1*-expressing (left) plants. Bar = 1 cm.

**Figure 9:**
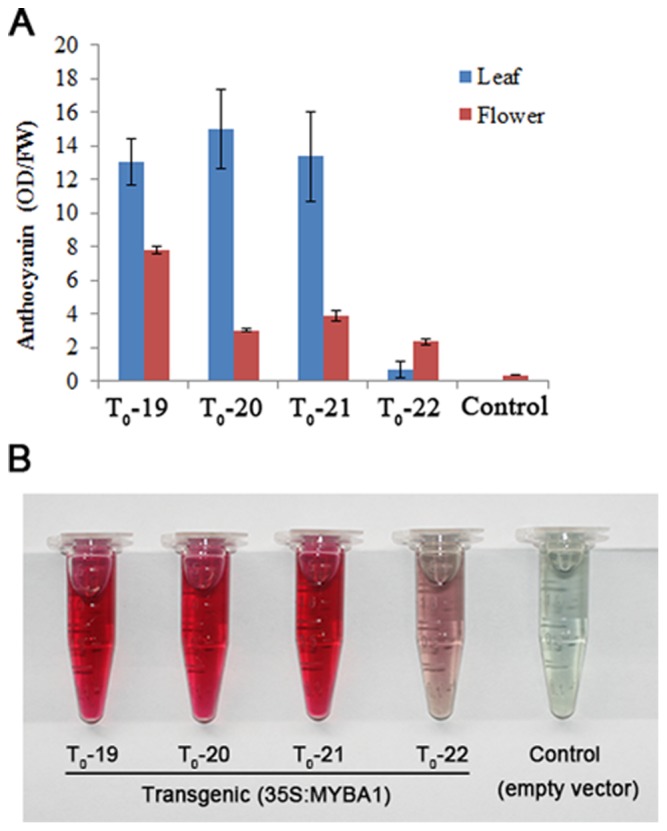
Total anthocyanin content measurement from flowers and leaves of transgenic tobacco plants overexpressing *EsMYBA1* and empty vector (control). (A) Total anthocyanin contents of flowers and leaves were measured from four transgenic tobacco lines (T_0_-19 to T_0_-22) overexpressing *EsMYBA1* and control line expressing empty vector. Each column represents the mean value with error bar indicating SD from three technical replicates for each sample. Each sample of flowers was collected from three whole flowers, and each sample of leaves was harvested from three leaves that are fourth leaf from the top at the blooming stage of tobacco. (B) Anthocyanin extracts from leaves of four transgenic tobacco lines (T_0_-19 to T_0_-22) overexpressing *EsMYBA1* and control line expressing empty vector.

To investigate the function of *EsMYBA1*, we overexpressed the *EsMYBA1* gene driven by the CaMV 35S promoter, in tobacco and *Arabidopsis*. Ectopic expression of *EsMYBA1* induced strong anthocyanin accumulation in the vegetative (Figure 8A–C) and reproductive tissues (Figure 8D–H) of transgenic tobacco. The whole flower of overexpression transgenic lines, including sepal, petal, anther, filament and pistil, exhibited dark-red pigments compared with control lines expressing the empty vector (Figure 8I–L). Capsule skin from overexpression lines displayed black-red color, in which the immature seed coat showed black-purple color (Figure 8K, L), although no distinct color change from the control line was observed in the mature seed coat (Figure 8M). In addition to the color change, most of the overexpression lines showed stunted or delayed phenotypes compared with the control line (Figure 8A). Total anthocyanin content was significantly higher in the flowers of four overexpression transgenic tobacco lines (T_0_-19 to T_0_-22) than that of the control line, and a similar result was also observed in leaves of transgenic tobacco lines (Figure 9A). Remarkably, in three of four overexpression lines (T_0_-19 to T_0_-21), anthocyanin content was higher in leaves than in flowers (Figure 9A). Moreover, the color of anthocyanin extraction from transgenic tobacco leaves reflected the total anthocyanin level (Figure 9B).

### Up-regulation of most Flavonoid Biosynthetic Genes in Transgenic Tobacco and *Arabidopsis* with *EsMYBA1* Overexpression

**Figure 10:**
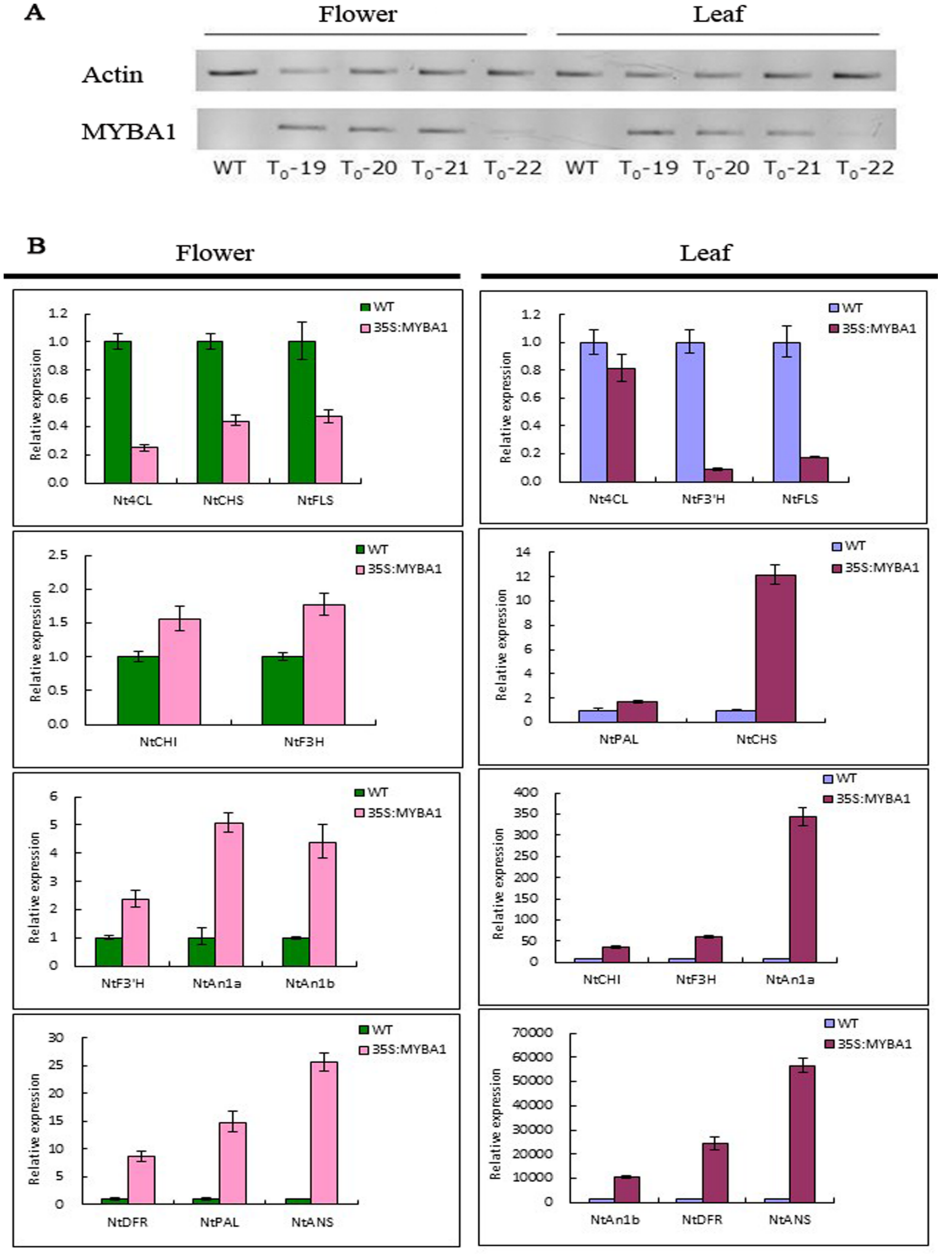
Quantitative RT-PCR analysis of transcription levels of the flavonoid pathway genes in transgenic tobacco plants carrying empty vector as control (WT) and *EsMYBA1* gene. (A) Semi-quantitative RT-PCR assay was used to confirm the *EsMYBA1* expression in the flowers and leaves of transgenic tobaccos plants, and the *Actin* gene from tobacco was selected as a positive control. (B) Quantitative RT-PCR assay was used to determine the relative levels of nine structural genes and two *bHLH* regulators of the flavonoid pathway in the transgenic tobacco flowers and leaves, including *PAL*, *4CL*, *CHS*, *CHI*, *F3H*, *F3’H*, *FLS*, *DFR*, *ANS*, *AN1a* and *AN1b*. The tobacco *Tub1* gene was used as an internal control, and the comparative Ct method was used to determine the relative level, while the expression level of gene in the control lines was set to “1″. The column shows the average value with SD bar from three technical replicates.

**Figure 11:**
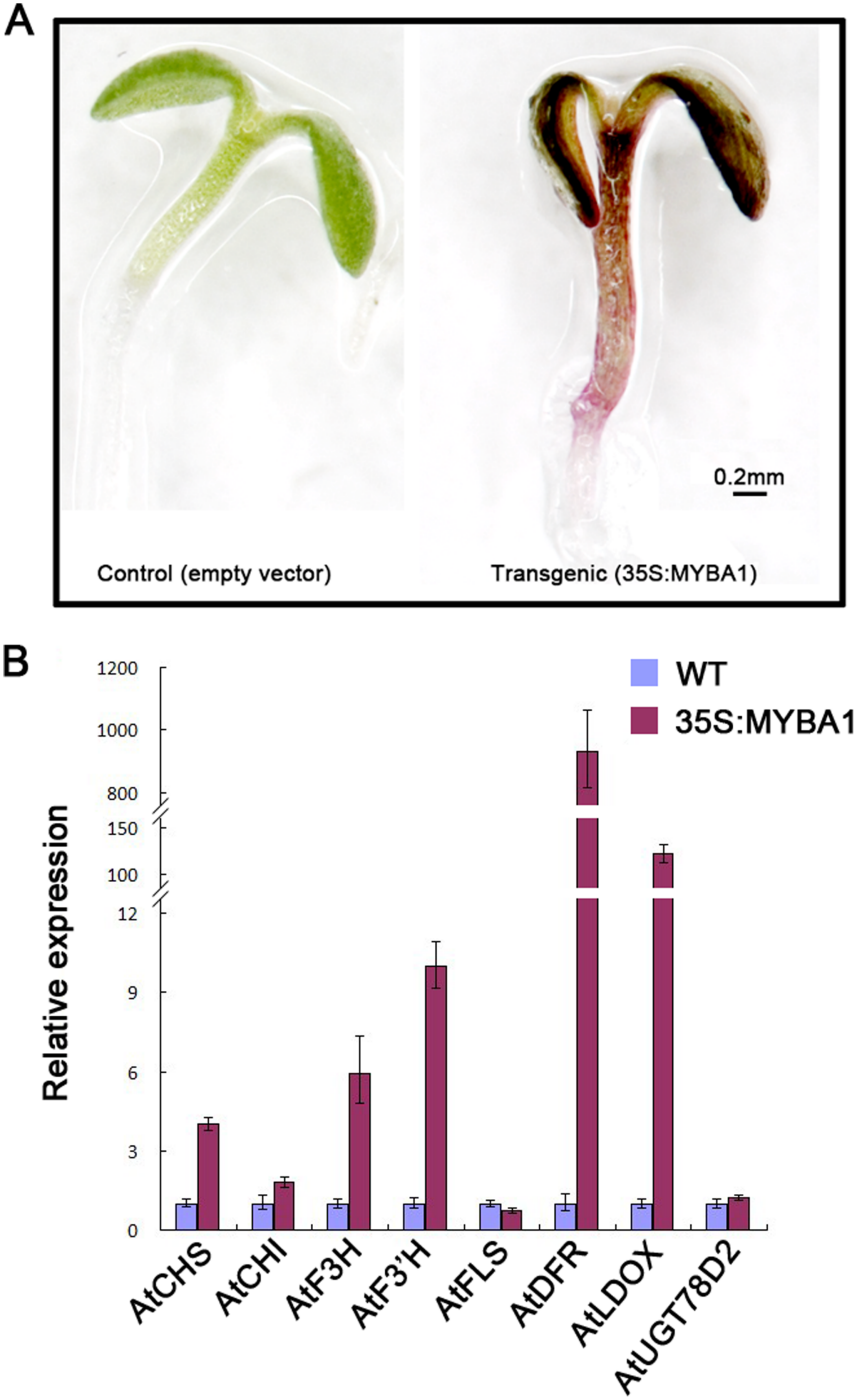
Phenotype observation and quantitative RT-PCR analysis of the transgenic *Arabidopsis* plants overexpressing *EsMYBA1* and empty vector (control). (A) Color change occurred in the transgenic *Arabidopsis* seedlings (right), which showed the red pigments compared to the control plants (left). (B) Quantitative RT-PCR analysis of transcription levels of the flavonoid biosynthetic pathway genes in the transgenic *Arabidopsis* plants overexpressing *EsMYBA1* and empty vector as control (WT). Eight structural genes of anthocyanin biosynthetic pathway were selected for analysis, including *CHS*, *CHI*, *F3H*, *F3’H*, *FLS*, *DFR*, *LDOX* and *UGT78D2*. The *Arabidopsis TUB2* gene was used as an internal control, and the comparative Ct method was used to determine the relative level, while the expression level of gene in the control lines was set to “1″. The column shows the average value with SD bar from three technical replicates.

QPCR analysis was performed to examine the effect of the introduced *EsMYBA1* on the endogenous flavonoid pathway genes in tobacco and *Arabidopsis*. Expression of the *EsMYBA1* gene in tobacco flowers and leaves was first confirmed by semi-quantitative RT-PCR. *EsMYBA1* was expressed in both flowers and leaves from the four overexpression transgenic tobacco lines, but not in the negative control expressing the empty vector (Figure 10A). Compared with the control line, most of the structural genes of the flavonoid biosynthetic pathway, including *NtPAL* (phenylalanine ammonia-lyase), *NtCHI* (chalcone isomerase), *NtF3H* (flavanone 3-hydroxylase), *NtDFR* and *NtANS*, were up-regulated in both flowers and leaves of the overexpression transgenic tobacco lines. In particular, *NtDFR* and *NtANS* showed higher levels of up-regulation. Moreover, two regulatory *bHLH* TFs (*NtAN1a* and *NtAN1b*) were also noticeably up-regulated, especially in transgenic tobacco leaves (Figure 10B). In addition, down-regulation of *Nt4CL* (4-coumarate-CoA ligase) and *NtFLS* (flavonol synthase) was observed in both flowers and leaves of the overexpression lines (Figure 10B). However, the expression changes of two structural genes differed in flower and leaf. *NtCHS* (chalcone synthase) was down-regulated in transgenic flowers, but up-regulated in transgenic leaves. *NtF3’H* (flavonoid 3′-hydroxylase), on the other hand, displayed the opposite expression pattern from *NtCHS* (Figure 10B). Similar to the transgenic tobacco plants, anthocyanin accumulation was also strongly induced in seedlings of transgenic *Arabidopsis* overexpressing the *EsMYBA1* gene (Figure 11A). QPCR analyses of flavonoid genes in *Arabidopsis* seedlings overexpressing *EsMYBA1* revealed similar results as observed in the transgenic tobacco plants (Figure 11B). With an exception of *AtFLS* gene, most flavonoid genes were induced, in particular, *AtDFR* and *AtLDOX* (leucoanthocyanidin dioxygenase) were increased more than 900-fold and 150-fold, respectively.

### Anthocyanin Accumulation in Epimedium Leaves with Transient Expression of *EsMYBA1*

**Figure 12:**
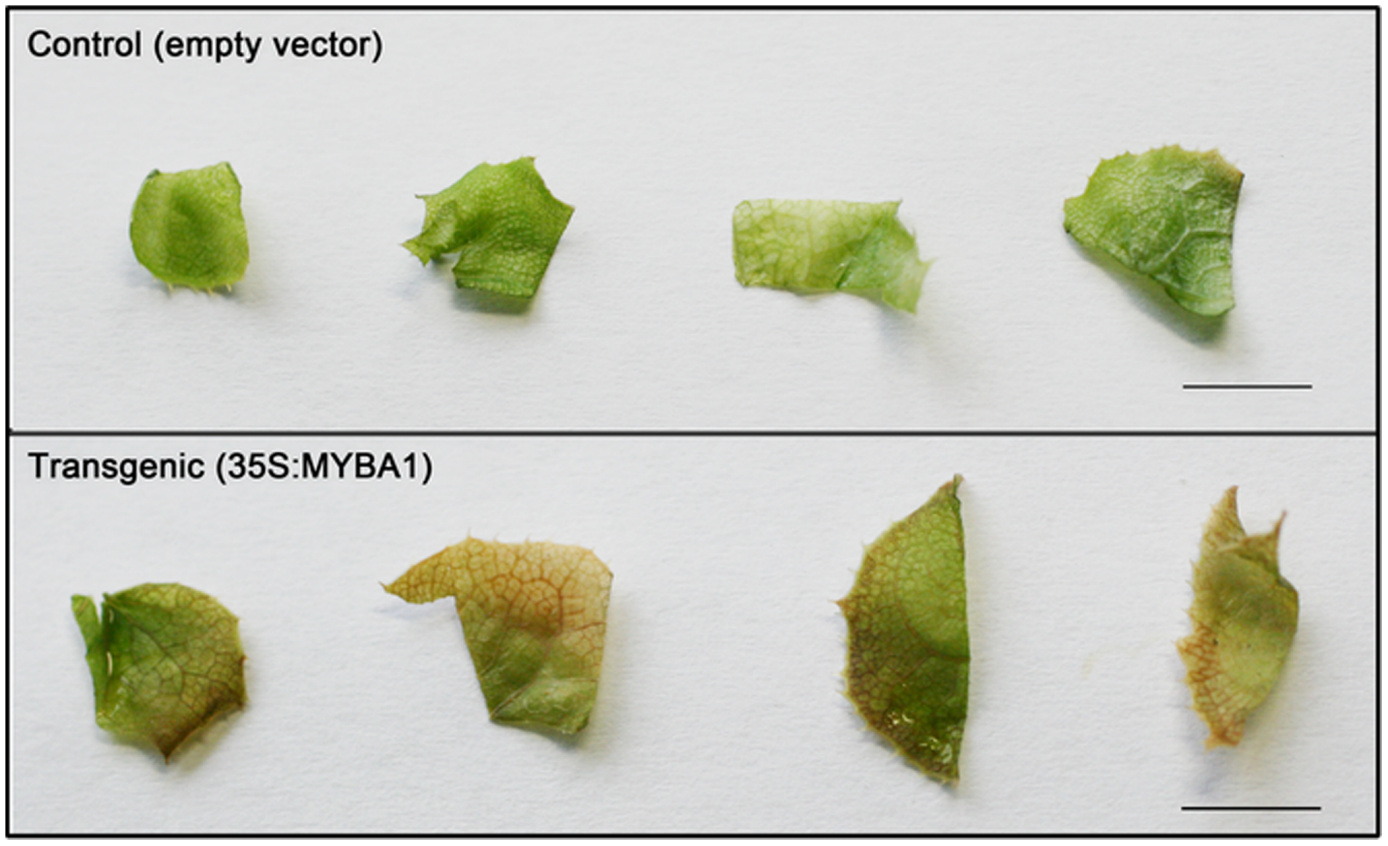
Transient expression of *EsMYBA1* in the leaves of Epimedium *in vitro*. Young leaves were excised from sterile cultured plantlets of *E. sagittatum* and co-cultured with *Agrobacterium* strain EHA105 carrying the *EsMYBA1* gene under control of the CaMV 35S promoter (35S:EsMYBA1) or the empty vector (control), and the photos were taken after 3 days by digital camera. Bar = 1 cm.

The strong induction of anthocyanin accumulation induced by *EsMYBA1* was further validated by transient expression of *EsMYBA1* in *E. sagittatum* leaves. The 35S:EsMYBA1 construct used for overexpression in tobacco and *Arabidopsis* was transformed by agro-infiltration for transient expression in excised leaves in sterile culture from *E. sagittatum*. After two days of co-culture, red pigments were observed mainly in the wounded area of transgenic leaves while no visible color change occurred in control leaves expressing the empty vector (Figure 12). These results suggest that overexpression of *EsMYBA1* in Epimedium can also induce anthocyanin accumulation in leaves.

## Discussion

### *EsMYBA1* is Homologous with other *R2R3-MYB* Genes Involved in Regulation of the Anthocyanin Biosynthetic Pathway

The high level of sequence homology and close phylogenetic relationship shared by EsMYBA1 and a number of R2R3-MYB regulators of the anthocyanin pathway suggest that EsMYBA1 is likely to be involved in regulation of the anthocyanin biosynthetic pathway. The presence of three conserved motifs associated with anthocyanin biosynthesis related MYB TFs in the C-terminal region of EsMYBA1 also suggests that EsMYBA1 is a strong candidate as a key MYB regulator of the anthocyanin pathway (Figure 1A). The R2R3-MYB family from *Arabidopsis* is divided into 24 subgroups based on conserved residues present outside MYB domains. The R2R3-MYB subgroup 6 that is involved in the anthocyanin pathway regulation, including PAP1 and PAP2, has the conserved motif KPRPR[S/T]F [13]. Many other anthocyanin-related R2R3-MYB regulators, such as IbMYB1 [20], MdMYB10 [21], MrMYB1 [57], MtLAP1 [24] and NtAN2 [47], also contain this subgroup 6-specific motif, although the function of this motif remains unknown. Another conserved motif, [A/S/G]NDV, identified recently from a study on Rosaceae MYB10 to distinguish anthocyanin and non-anthocyanin MYB regulators in dicots [56], is also present in the EsMYBA1 sequence. In addition, phylogenetic analysis shows that EsMYBA1 is closest to MtLAP1, from *Medicago*, and located in the basal position of the anthocyanin-promoting MYB clade (Figure 1B). This is consistent with the placement of two *R2R3-MYB* genes, *LhMYB6* and *LhMYB12* from lily, also in the basal position of the *AN2* subgroup [58].

### Conserved Genomic Structure and Alternative Splicing of *EsMYBA1*

The genomic structure of *EsMYBA1* shows an orthologous relationship with anthocyanin-promoting *MYB* genes. Moreover, this exon/intron organization is conserved among several characterized anthocyanin-related *R2R3-MYB* genes, such as *Arabidopsis AtPAP1* [15], tobacco *NtAN2* [47], sweet potato *IbMYB1* [20] and alfalfa *MtLAP1* [24]. The high degree of sequence similarity and conserved genomic structure suggests that these anthocyanin-related MYB genes may be derived from a common evolutionary origin. It is noteworthy that the alternative splicing of *EsMYBA1* results in three different transcripts, of which two intron-retaining transcripts encode two open reading frames for MYB-related proteins (Figure 2). Presently, the function of these MYB-related proteins from Epimedium is unknown. It has been demonstrated that MYB proteins with a single repeat are involved in many biological processes, such as epidermal patterning [59] and anthocyanin biosynthesis [38], [39]. Alternative splicing for *R2R3-MYB* TFs has been described previously for maize *P*, which encodes two transcripts that are alternatively spliced at the 3′ ends [60], and rice *myb7*, which contains both spliced and unspliced forms, with splicing being enhanced by anoxia [61], [62]. Recently, it was found alternative splicing in *AtMYB59* and *AtMYB48*, from *Arabidopsis*, and the two rice homologues, *OsMYBAS1* and *OsMYBAS2*, produces two types of MYB-related or R2R3-MYB proteins [63]. We show here, *EsMBA1* also appears to possess this ability, leading to two MYB-related proteins, in spite of their unknown functional relationship. The shorter transcript of maize *P* has been suggested to act as a competitive inhibitor of the functional *P* protein [60]. The MYB-related proteins from *EsMYBA1* may also act in a similar fashion as a negative inhibitor of the functional EsMYBA1 protein by disrupting the MBW complex.

### *EsMYBA1* Preferentially Expresses in Leaves

Many *R2R3-MYB* regulators of the anthocyanin biosynthetic pathway are abundantly expressed in anthocyanin-rich tissues and correlate strongly with anthocyanin accumulation [16], [21], [47], [57], [64]. For example, the expression of *LhMYB6* and *LhMYB12* corresponded well with anthocyanin pigmentation in various tissues [58]. When examined using green-leafed Epimedium, *EsMYBA1* expression in various tissues showed no strong correlation with anthocyanin accumulation. The preferential expression of *EsMYBA1* in leaves may be associated with accumulation of main bioactive compounds. Flavonoids, the main bioactive components of Epimedium, accumulate abundantly in leaves and are perhaps involved in protecting plants against UV light and pathogen attack [42]. We suggest that *EsMYBA1* has a broad function, which not only regulates anthocyanin biosynthesis, but also biosynthesis of other flavonoids, such as flavonols. When constitutively expressed in transgenic alfalfa, *MtLAP1* induces, not only massive accumulation of anthocyanin pigments, but also PA-like compounds in leaves [24]. However, when comparing the green-leafed and red-leafed Epimedium, *EsMYBA1* expression correlated well with anthocyanin accumulation in leaves. The expression level of *EsMYBA1* is far higher in red leaves that accumulate more anthocyanin than green leaves (Figure 3E–F). In addition, we have isolated another *R2R3-MYB* gene (designated as *EsAN2*) which shows high level of homology of *PhAN2*. *EsAN2* is mainly expressed in the anthocyanin-rich tissues, including flower buds and flowers (unpublished data), which suggests that *EsAN2* may be a key factor controlling anthocyanin accumulation in floral tissues. The preferential expression of the *AN2* gene in floral tissues from petunia and tobacco corresponds well with the expression pattern of *EsAN2* [16], [47]. Within the *AN2 R2R3-MYB* subgroup, two or more genes are often present in a single plant species, such as *PAP1*, *PAP2*, *AtMYB113* and *AtMYB114* in *Arabidopsis* [65], *AmROSEA1* and *AmROSEA2* in snapdragon [66], *VlMYBA1* and *VlMYBA2* in grape [19], and *LhMYB6* and *LhMYB12* in lily [58]. These results suggest that *EsMYBA1* and *EsAN2* probably regulate the anthocyanin biosynthesis and determine tissue-specific accumulation of anthocyanin in Epimedium.

### Anthocyanin Production in both Vegetative and Reproductive Tissues of Tobacco and *Arabidopsis* with Ectopic Expression of *EsMYBA1*

Overexpression of anthocyanin-related *MYB* regulators often leads to enhance anthocyanin accumulation in heterologous or homologous plant species [15], [47], [64]. In this study, when constitutively expressed in tobacco, *EsMYBA1* induced massive accumulation of anthocyanin in both reproductive and vegetative tissues, particularly stamen and pistil tissues showing dark red color (Figure 8). In addition, a high amount of anthocyanins accumulated in the capsule skin and immature seed coat, while no obvious color change is observed in the mature seed coat compared to the control (Figure 8). However, transgenic *Arabidopsis* and tobacco, overexpressing *NtAN2*, produced darker seeds because of increased anthocyanin accumulation, rather than PA, in the seed coat [47]. The oxidation of PAs during the course of seed desiccation leads to the formation of brown pigments that confer color to the mature seed [67], and this brown color of PAs possibly interferes with the red color of anthocyanin pigments.

In addition to reproductive tissues, dramatic increases in anthocyanin production are also observed in the vegetative tissues of the transgenic tobacco (Figure 8). During early developmental stages, kanamycin-resistant shoots overexpressing *EsMYBA1* show red pigments (data not shown). The mature transgenic plants are clearly darker, close to purple color, and significant amounts of anthocyanin can be extracted from leaves (Figure 8; Figure 9). A similar phenotypic change was reported on transgenic alfalfa plants overexpressing *MtLAP1*, which accumulate large amounts of anthocyanin in vegetative tissues, including leaves, stems, and even roots [24]. In transgenic *Arabidopsis*, *EsMYBA1* overexpression also induces anthocyanin accumulation in seedlings (Figure 11A), which is consistent with reports on *IbMYB1* overexpression analysis [20]. We are also interested in functionally validating *EsMYBA1* in Epimedium cells. Due to currently the lack of method for stable transformation of Epimedium, we transiently expressed *EsMYBA1* in leaves of Epimedium. Many visible red pigment spots were observed in the wounded area (Figure 12), suggesting *EsMYBA1* also probably induces anthocyanin accumulation in Epimedium leaves. The results from both transient and stable transformation experiments indicate that *EsMYBA1* has a conserved function of regulating anthocyanin accumulation. In addition, the relative level of *EsMYBA1* expression positively correlates with anthocyanin production in transgenic tobacco. Semi-quantitative RT-PCR analysis of four transgenic lines with different levels of total anthocyanin indicates that higher *EsMYBA1* expression leads to more anthocyanin accumulation (Figure 9A; Figure 10A). A similar correlation between mRNA levels and anthocyanin production has been shown for tobacco *An2* [47] and apple *MYB10* [21].

### Expression of Flavonoid-related Genes Affected by *EsMYBA1* in Transgenic Tobacco and *Arabidopsis*

Anthocyanin accumulation is strongly enhanced in transgenic tobacco and *Arabidopsis* plants overexpressing *EsMYBA1*, suggesting that the structural genes of the anthocyanin pathway must be affected. Most structural genes of the flavonoid biosynthetic pathway were up-regulated in both transgenic tobacco and *Arabidopsis*; most noticeably, the expression of *DFR* and *ANS* were greatly enhanced (Figure 10B; Figure 11B). Because transient luciferase assay experiments validate that EsMYBA1 can bind to both *DFR* and *ANS* promoters of Epimedium, tobacco and *Arabidopsis* (Figure 7), these results imply that *EsMYBA1* can directly regulate the same subsets of genes in all three species. Anthocyanin branch genes can be divided into two subsets: early genes (*CHS*, *CHI*, and *F3H*) and late genes (*DFR* and *ANS*) [65]. The *MYB* regulators can regulate either the early or late genes or both. Expression of sweet potato *IbMYB1* or alfalfa *MdLAP1* induces both early and late genes of the anthocyanin pathway in transformed plants [20], [24], while the maize MYB protein P1 regulates only the early, but not late genes [68]. In both transgenic tobacco and *Arabidopsis* plants overexpressing *EsMYBA1*, the *FLS* gene was down-regulated (Figure 10B; Figure 11B). This is possibly because the main metabolic flux of the flavonoid pathway is directed from the flavonol branch to the anthocyanin branch. FLS, as the first enzyme of the flavonol biosynthesis pathway, is located at the branching point between anthocyanin and flavonol pathways, and competes with DFR for the same dihydroflavonol substrate. We surmise that the strong up-regulation of *DFR* leads, in part, to the reduction of *FLS* expression. Determination of flavonoid composition and content in transgenic plants will be needed to further validate this supposition. Nevertheless, we conclude that *EsMYBA1* expression can regulate both the early and late genes of the anthocyanin biosynthetic pathway.

### Interaction of EsMYBA1 and bHLH TFs Involved in Regulation of the Flavonoid Pathway

R2R3-MYB TFs are well established to interact with bHLH TFs to regulate the flavonoid pathway in plants [9], [65]. In tobacco, NtAN2, a MYB protein, interacts with bHLH regulator NtAN1 to regulate the anthocyanin biosynthesis in floral tissues [47], [48]. In yeast cells, we have demonstrated that EsMYBA1 interacts with NtAN1a and NtAN1b, as well as several other bHLH TFs (Figure 5). A similar result has been described previously, showing that NtAN2 is capable of interacting with some other heterologous Lc-Like bHLH proteins [47]. The interaction between EsMYBA1 and NtAN1a is confirmed further by BiFC assay in *Arabidopsis* protoplasts (Figure 6). In transgenic tobacco, both *NtAN1a* and *NtAN1b* are strongly activated in leaves and flowers by *EsMYBA1* expression (Figure 10B). This is consistent with reports that overexpression of *NtAN2* can induce expression of both *NtAN1a* and *NtAN1b* [48]. *NtAN1* is not normally expressed in tobacco leaves where the anthocyanin pathway is inactive. However, many reports have showed that overexpression of several *bHLH* regulators, including perilla *Myc-RP* in tobacco, maize *Lc* in *Arabidopsis* and tobacco, snapdragon *Delila* in tomato and tobacco, as well as *NtAN1* in tobacco, result in enhanced pigmentation that is restricted to tissues that are normally pigmented in wild types [48], [69]–[71]. The lack of anthocyanin production in transgenic tobacco leaves is possibly due to the fact that *NtAN2* is not activated in tobacco leaves. Like *NtAN2* regulation of *NtAN1* expression [48], *EsMYBA1* can also activate *NtAN1* and then interact with *NtAN1* to induce the expression of key structural genes, resulting in enhanced anthocyanin accumulation in both leaves and flowers of tobacco. In addition, EsMYBA1 also induces anthocyanin accumulation in *Arabidopsis* seedlings (Figure 11A). The MYB/bHLH/WD-repeat complex is well-established as a regulator of the phenylpropanoid pathway in *Arabidopsis* [65], thus it is likely that *AtTT8* can be activated by *EsMYBA1* expression in *Arabidopsis*, because the interaction between EsMYBA1 and AtTT8 is confirmed by both Y2H and transient luciferase assay (Figure 5; Figure 7). In addition to the interaction between EsMYBA1 and heterologous bHLH TFs from other plant species, the interaction between EsMYBA1 and EsTT8 was supposed, based on result of the transient luciferase assay (Figure 7). Combined with that *EsMYBA1* regulates the two subsets of the anthocyanin biosynthetic genes, these facts provide an explanation as to why the transient expression of *EsMYBA1* results in anthocyanin accumulation in Epimedium leaves.

In conclusion, we here described a *R2R3-MYB* TF, *EsMYBA1*, isolated from *E. sagittatum*. *EsMYBA1* is the first *R2R3-MYB* gene to be functionally characterized in Epimedium, and is involved in regulating the flavonoid biosynthetic pathway. The isolation and characterization of *EsMYBA1* opens a door for understanding and engineering the accumulation pattern of anthocyanin contributed to the colorful flower and leaf, and of flavonoids contributed to the main bioactive compound in Epimedium.

## Supporting Information

File S1 **Table S1.** List of primers used for *EsMYBA1* isolation and characterization. **Table S2.** List of primers used for qPCR assay in transgenic tobacco. **Table S3.** List of primers used for qPCR assay in transgenic *Arabidopsis thaliana*.
(DOCX)
