# Supplementary material for: A R2R3-MYB Transcription Factor from Epimedium sagittatum Regulates the Flavonoid Biosynthetic Pathway
Source: PLoS One. 2013 Aug 1;8(8):e70778. doi: 10.1371/journal.pone.0070778 (PMC3731294; doi:10.1371/journal.pone.0070778)
Supplement: File S1 — Table S1. List of primers used for EsMYBA1 isolation and characterization. Table S2. List of primers used for qPCR assay in transgenic tobacco. Table S3. List of primers used for qPCR assay in transgenic Arabidopsis thaliana. (DOCX) [file pone.0070778.s001.docx]

Table S1 List of primers used for *EsMYBA1* isolation and characterization

| Name | Forward primer (5’- 3’) | Reverse primer (5’-3’) | Note |
| --- | --- | --- | --- |
| Degenerate primers for *MYB* cDNA | AARTAYGGNGARGGNAARTGGCA | CCARTARTTYTTNACRTCRTTNGC |  |
| Full-length *EsMYBA1* cDNA and gDNA | TAGAGAACTTAAATGAAGCCAGAT (1.F) **^a^** | AGAAAACAATGCAAAAATAGAATC (1.R) |  |
| ORF of *EsMYBA1* for subcellular localization | GCGGATCCATGAAGCCAGATTTTAGTGAGAT **^b^** | CGGTCGACTTCAAAATTCCAAAAGTTCAAG |  |
| ORF of *EsMYBA1* for BiFC assay | GCCTCGAGATGAAGCCAGATTTTAGTGAGAT | CGGGATCCTTCAAAATTCCAAAAGTTCAAG |  |
| Full-length *EsMYBA1* for transient luciferase assay | ATGAAGCCAGATTTTAGTGAGAT | AGAAAACAATGCAAAAATAGAATC |  |
| Full-length *EsTT8* cDNA | TAATTGGGTTCCGGTAGAAAGAAT | TACAGTCAATCACATTATATACCAACACT |  |
| Full-length *EsTT8* for transient luciferase assay | GTGCAACCCATGGAGGTAAGT | TACAGTCAATCACATTATATACCAACACT |  |
| Full-length *AtTT8* for transient luciferase assay | GTATCTCCGGGAACGATGGATG | TTGGCATCAATAAAGTTAGGGTCTA |  |
| *EsCHS* promoter sequence | ACATGTGTGGATTTGGCTTAACG | TTAGCTCTTACTGTTATTATTTATCACG | Used for transient luciferase assay |
| *EsDFR* promoter sequence | GATGTTTTTCTATGTCGGTCTCTAT | GCTATATTTTCTTCAAGCTTTTCT |  |
| *EsANS* promoter sequence | ACGAGTTGGGGATTACTG | GGTTACAAAAACAGATTTTCTCTTG |  |
| *AtDFR* promoter sequence | GAGATTGGCACCACCTTCGCCTC | TTTTGTGGTTATATGATAGATTGTGCT |  |
| *NtDFR* promoter sequence | GCTCATAATGACTCGATTACG | CAGAAATGAAAGGTAGAAGAG |  |
| *EsMYBA1* alone for qPCR assay | TCAAAGGGCAGGTCTGAATCG (q1.F) | TCAGTGACCATCTGTTTCCGAG (q1.R) |  |
| *EsMYBA1.1* for qPCR assay | AATACCTCAAAGGGCAGGCAAG (q1.1.F) | CAGACCTTCAAAACCATAAGCCAT (q1.1.R) |  |
| *EsMYBA1.2* for qPCR assay | AGGCAAGTTTCCAGCTATCGTG (q1.2.F) | TTCCCGCAATCAGTGACCATC (q1.2.R) |  |
| *EsMYBA1* total for qPCR assay | TGGTCGGACAGCCAATGATG (q1.total.F) | AGGGGGTAGTATCCTTTCCTTGTC (q1.total.R) | The fragment shared by three transcripts of EsMYBA1 gene |
| Intron primers for alternative splicing | CTATCGTGCTGATTTTTTCTGTATTC (Intron II.F) | CCACAAAAGTAGTTCAACATGCAAC (Intron I.R) | RT-PCR for alternative splicing |
| *EsActin* for qPCR assay | GCCATTCAGGCTGTTCTTTC | GGTAAGATCGCGACCTGCTA |  |
| *NtActin* for RT-PCR assay | TAAGCAACTGGGACGATATG | CAAGATCCAACCGAAGAAT |  |

^a^ indicates primer names in parenthesis used for genomic structure and alternative splicing of *EsMYBA1*

^b^ indicates the additional restriction enzyme site are underlined

Table S2 List of primers used for qPCR assay in transgenic tobacco

| Gene name | Accession number | Forward primer (5’-3’) | Reverse primer (5’-3’) | PCR length |
| --- | --- | --- | --- | --- |
| *NtPAL* | X78269 | CGATAGACTTGAGGCATTTG | AGGTTCTCTTAGCGACTTG | 78 |
| *Nt4CL* | U50845 | GCGACATTGGGTTCATTG | TTCTCCTGCTTGCTCATC | 176 |
| *NtCHS* | AF311783 | AGCGAGCATAAGGTTGAG | ACCACCACTATGTCTTGTC | 164 |
| *NtCHI* | AB213651 | CTTTTCTCGCCGCTAAATG | TTTCTGCCACCTTCTCTG | 159 |
| *NtF3H* | AB289450 | GAGGCAATGGGCTTAGAG | TCAGTGTGTCGTTTCAGTC | 128 |
| *NtF3'H* | AB289449 | AGCCATAGTCAAGGAAACC | CTCACAACTCTCGGATGC | 79 |
| *NtDFR* | EF421429 | TAAGAAGATGACAGGATGGATG | TGGCGGTATGATGCTAATG | 109 |
| *NtFLS* | DQ435530 | GTCCCATATAACCATTCTTGTC | CACTCTTGTATTTCCCATTGC | 152 |
| *NtANS* | AB289447 | CTACATTCCAGCAACAAGTG | GTCCCAGCCCAATAGAAAG | 86 |
| *NtAn1a* | HQ589208 | ACCATTCTCGAACACCGAAG | TGCTAGGGCACAATGTGAAG | 97 |
| *NtAn1b* | HQ589209 | CTTGAACACTTCTCAAACCGA | TGCTAGGGCACAATGTGAAG | 100 |
| *NtTub1* | AJ421411 | TCCGTGGTGATGTTGTG | TGGTGGCTGATAGTTGATAC | 125 |

Table S3 List of primers used for qPCR assay in transgenic *Arabidopsis thaliana*

| Gene name | Gene identifier | Forward primer (5’-3’) | Reverse primer (5’-3’) | PCR length |
| --- | --- | --- | --- | --- |
| *AtCHS* | AT5G13930.1 | CGAGATGAGGAGGAAGTC | GGTAGGTAGGCAGATAGAAG | 163 |
| *AtCHI* | AT2G26310.1 | GTTATGAGTGTGAGGCTTG | GGGTATTGGAATGTCTTGTC | 168 |
| *AtF3H* | AT3G51240.1 | GTATAAGAGAAAGATGGGAAGAG | CAACAACAAGTAAGCAAGAAC | 142 |
| *AtF3'H* | AT5G07990.1 | TTGATGTGAAAGGAAGCG | GCCGTAAGAAACTGAATCG | 103 |
| *AtFLS* | AT5G08640.1 | CCTAAGAATCCACCTGAATAC | CCCTAATCCATCCGAGAG | 102 |
| *AtDFR* | AT5G42800.1 | GCAACCATTCTTACTATCTCC | TCTCATCAACACCTTCAAAC | 85 |
| *AtLDOX* | AT4G22880.1 | ACTTCTTTCATCTTGCGTATC | TCGTTGCTTCTATGTAATCAC | 84 |
| *AtUGT78D2* | AT5G17050.1 | TCTCTGCTCATCTCTACAC | CTCCATACGCTCACCTAC | 71 |
| *AtTUB2* | [AT5G62690.1](http://www.arabidopsis.org/servlets/TairObject?id=135732&type=gene) | TGGCATCAACTTTCATTGGA | ATGTTGCTCTCCGCTTCTGT | 157 |
